# The Nutrition of the External Table of the Skull

**Published:** 1863-09

**Authors:** 


THE NUTRITION OF THE EXTERNAL TABLE OF THE SKULL. —*Editor Med. and Surgical Reporter:*—The following described accident has, it seems to me, such conclusive bearing on a rather mooted question, viz., the source of nutrition to the external table of the skull, that I am indued to write it out for the *Reporter*, under the impression that its perusal will prove neither uninteresting or useless.

Master B., a lad some seven years of age, was knocked down and kicked by a horse, the blow taking effect upon the frontal region, tearing up the scalp a distance of four inches, the periosteum being raised with its neighboring layers as clearly as though it had been done by the hand of a surgeon. Called immediately after the accident, I dressed the wound in the usual manner, approximating the parts and retaining carefully applied wax silk sutures, with cold water dressings.

On the seventh day, the sutures, of which there were five, being no longer of service, were removed, union by first intention having been nicely secured.

Two days after this, the boy, being at play in the yard, fell from the top of the fence, striking the imperfectly consolidated wound, disuniting it some two inches. Being recalled to the case, stitches were applied as before. The next day, being in the cellar, the little patient stumbled over a stick of wood, again injuring the parts. The injury from this last accident was so severe as to cause a slough, exposing a portion of the frontal bone quite the size of a dime piece. The angry appearance of surrounding parts contraindicating further active treatment for the present, mild applications were resorted to, and the parts allowed to heal. This was effected with the exposure of the dime-sized portion of os frontis.

Five weeks of perfect rest was now allowed, the cicatrical tissue becoming very firm and healthy looking, the exposed surface of bone presenting the appearance as if a thin film of blood had dried over it. At the end of this period I loosened up the cicatrix, pared the edges, and by careful manipulation brought the parts together, thus covering the exposed bone, confining them in position with the hare-lip pins and figure of eight suture. The third day these pins sloughed out, and the parts fell back to their original position. The patient was now put upon a highly stimulating diet, combined with a tonic of iron and quinia.

Strapping with the ordinary adhesive strips was now resorted to, but the weather being very hot and the patient perspiring freely, these had not sufficient tenacity to resist the action of the occipito-frontalis muscle. The next step was to shave the head to the vertex. Two strips of India rubber plaster were prepared, the one stretching below the wound across the forehead, the other above across the vertex; some half a dozen or more strips of the most adhesive isinglass plaster stayed these transverse strips from a vertical direction. After these had thoroughly dried, stitches were passed from the strip below to the strip above, and by carefully made traction the parts were gotten together. Examination each day showing that the wound was tending to gap a little, fresh stitches were daily reapplied, the parts thus being held together. Powerful local stimulation was kept constantly applied to the parts; thus a union was finally secured and the long exposed bone covered.

To-day, September 1st, three months after the accident, I have carefully re-examined the parts; everything presents the most healthy appearance. I am convinced that the bone beneath the cicatrix is as healthy as that of any other part.

QUERY.—Does not this case prove that the external plate is not dependent exclusively upon its periosteum for its nutrition?

J. E. GARRETSON, M. D.

*Philadelphia, Sept.* 1, 1863.

